# Retinal Oxygen Delivery, Metabolism, and Extraction Fraction during Long-Term Bilateral Common Carotid Artery Occlusion in Rats

**DOI:** 10.1038/s41598-020-67255-4

**Published:** 2020-06-25

**Authors:** Sophie Leahy, Shayan Farzad, Norman P. Blair, Mahnaz Shahidi

**Affiliations:** 1 Department of Ophthalmology, University of Southern California, Los Angeles, CA USA; 2 Department of Ophthalmology and Visual Sciences, University of Illinois at Chicago, Chicago, IL USA

**Keywords:** Experimental models of disease, Retina

## Abstract

Retinal functional, biochemical, and anatomical changes have been previously reported in long-term experimental permanent bilateral common carotid artery occlusion (BCCAO). The purpose of the current study was to investigate progressive reductions in retinal oxygen metabolism (MO_2_) due to inadequate compensation by oxygen delivery (DO_2_) and extraction fraction (OEF) after BCCAO. Twenty-nine rats were subjected to BCCAO and were imaged after 3 hours, 3 days, 7 days, or 14 days. Six rats underwent a sham procedure. Phosphorescence lifetime and blood flow imaging were performed in both eyes to measure retinal oxygen contents and total retinal blood flow, respectively. DO_2_, MO_2_, and OEF were calculated from these measurements. Compared to the sham group, DO_2_ and MO_2_ were reduced after all BCCAO durations. OEF was increased after 3 hours and 3 days of BCCAO, but was not different from the sham group after 7 and 14 days. Between 3 and 7 days of BCCAO, DO_2_ increased, OEF decreased, and there was no significant difference in MO_2_. These findings may be useful to understand the pathophysiology of retinal ischemia.

## Introduction

Retinal ischemia is implicated in many ocular diseases, including ophthalmic artery occlusions, retinal vascular occlusions, ocular ischemic syndrome, diabetic retinopathy, and glaucoma^1,2^. Permanent bilateral common carotid artery occlusion (BCCAO) is an established experimental method in animals that reduces, but does not eliminate, blood flow to the retina, as well as the brain^3^. It has been used to investigate retinal ischemia, and previous studies have shown that long-term BCCAO results in a suppression of b-wave amplitude of the electroretinogram (ERG)^4–6^ and permanent loss of pupillary light reflex (PLR)^7–10^. Furthermore, optic nerve degeneration^8^ as well as reductions of retinal ganglion cell (RGC) layer thickness^7,8,11^ and inner plexiform layer (IPL) thickness^7^ have been demonstrated after long-term BCCAO. However, one study^4^ conducted after 7 days of BCCAO reported no retinal thinning and an increase in thickness of the outer plexiform layer (OPL) presumably due to edema. Although vascular compensation has been shown to normalize cerebral blood flow (CBF) 3 weeks^12^ and 4 weeks^13^ after BCCAO, the effect of vascular compensation on total retinal blood flow (TRBF) has not been reported.

We have previously demonstrated that complete occlusion of the ophthalmic vessels followed by reperfusion results in reductions of total retinal blood flow (TRBF), oxygen delivery (DO_2_), and oxygen metabolism (MO_2_)^14^. Furthermore, we reported the effect of reductions in TRBF on DO_2_, MO_2_, and oxygen extraction fraction (OEF) immediately and after a few days of BCCAO^15–17^. However, there is lack of knowledge about alterations in DO_2_, MO_2_, and OEF due to long-term, incomplete reduction in TRBF (in our case long-term BCCAO), which is more relevant to clinical ischemic conditions than assessment of changes in these parameters immediately after complete loss of blood flow. With longer durations of ischemia, it is expected that more cells demise due to lack of sufficient oxygen, resulting in reduced MO_2_. It is not known to what extent compensation by increased DO_2_ or OEF can maintain MO_2_. The purpose of the current study was to test the hypothesis that long-term incomplete retinal ischemia by BCCAO causes progressive reductions of MO_2_ due to inadequate compensation by DO_2_ and OEF.

## Results

**Figure 1:**
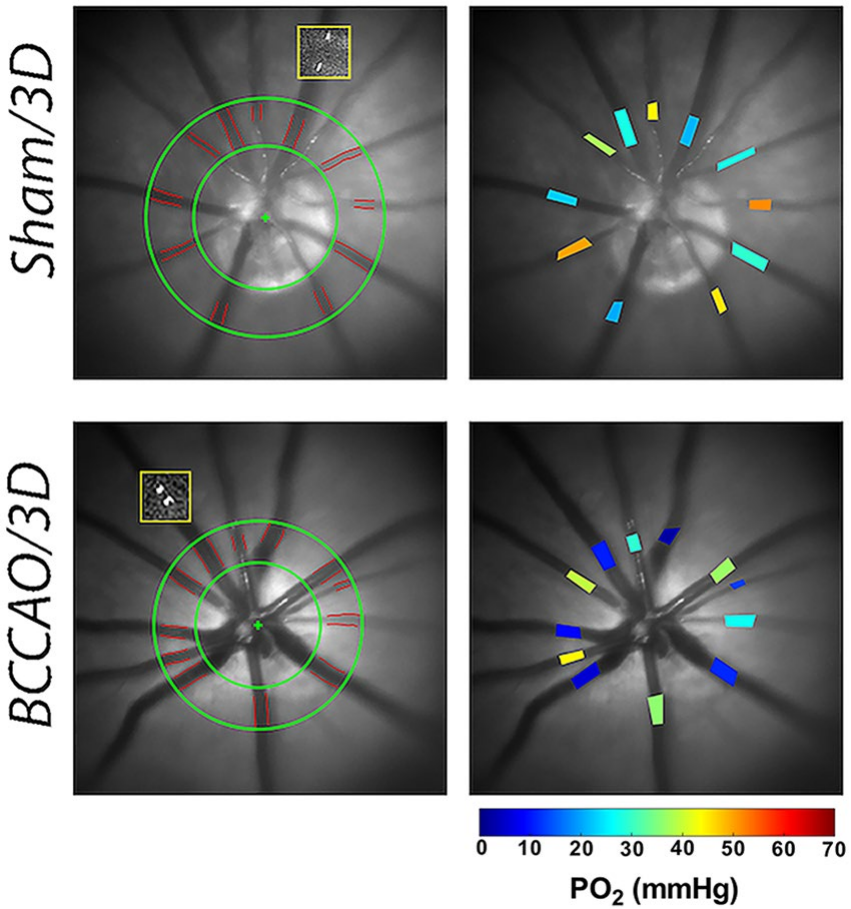
Technique of retinal vascular oxygen partial pressure (PO_2_) and blood flow imaging performed in sham and 3 days (3D) bilateral common carotid artery occlusion (BCCAO) groups. Red-free fundus images show the automatically detected retinal vessel boundaries outlined in red between green circles. Yellow boxes overlaid on the red-free fundus images show the intravenous microspheres at two time points. Reduced blood velocity can be observed in the rat from the 3 days BCCAO group compared to the rat from the sham group by the smaller distance the microsphere moved during the same time period. Retinal vascular PO_2_ measurements are presented in pseudo-color. Color bar shows PO_2_ values in mmHg.

Figure 1 shows examples of automatically detected vessel boundaries overlaid on red-free retinal images from eyes in the sham and 3 days BCCAO groups. For the same eyes, projection images generated by superimposing two images of one circulating fluorescent microsphere at 2 time points, 37 msec apart, depicts blood velocity. A larger distance between the positions of the microsphere indicated higher blood velocity (sham) compared to a smaller distance (BCCAO). Examples of retinal vascular oxygen partial pressure (PO_2_) measurements displayed in pseudo-color in the same eyes from the sham and 3 days BCAAO groups are shown in Fig. 1. Retinal arterial and venous PO_2_ in the eye from the sham group were higher than those in the eye from the 3 days BCCAO group.

### Diameter, velocity, and total retinal blood flow

**Figure 2:**
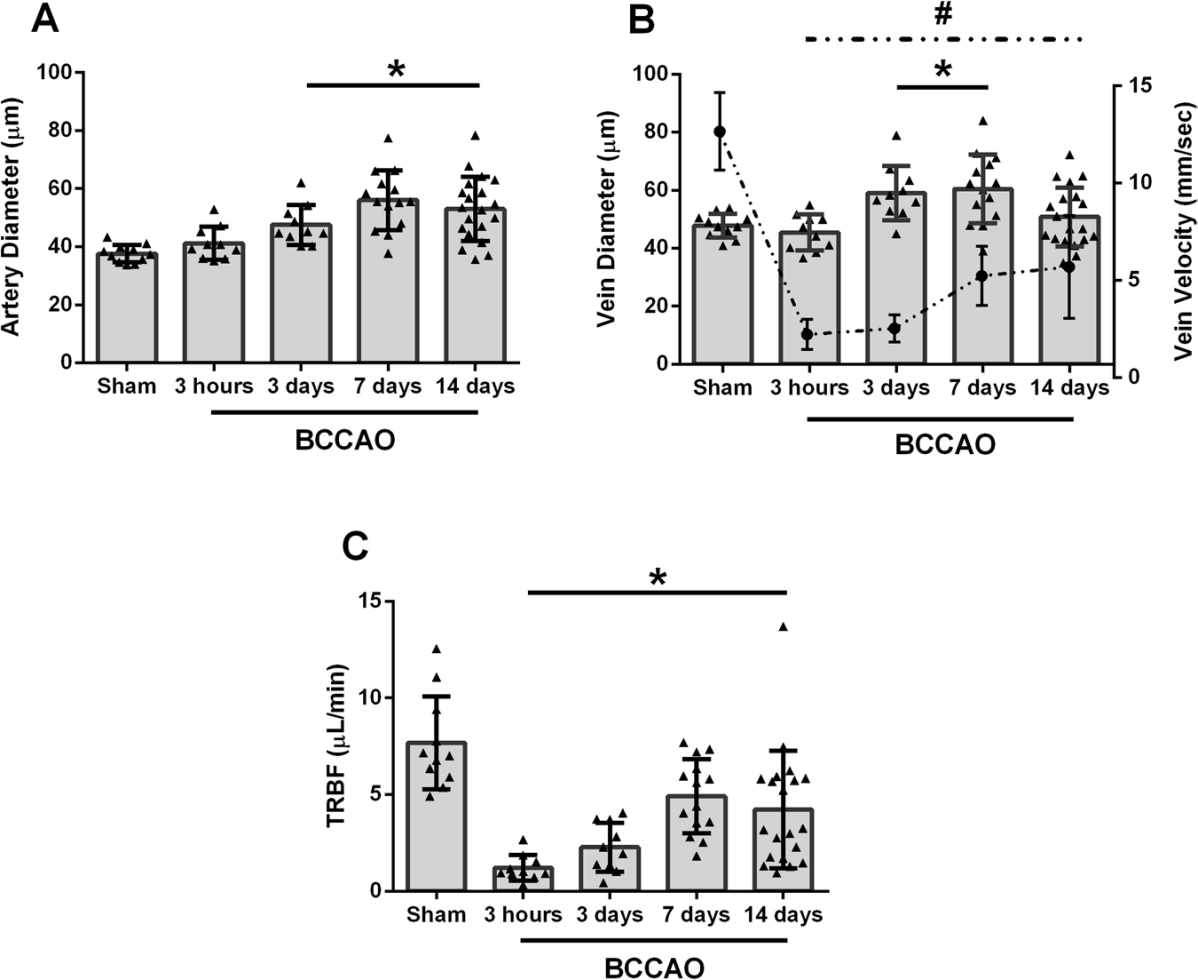
Comparison of arterial diameter (D_A_), venous diameter (D_V_), venous velocity (V_V_), and total retinal blood flow (TRBF) measured 3 hours, 3 days, 7 days, and 14 days after bilateral common carotid artery occlusion (BCCAO) as well as in sham group. Error bars indicate standard deviations. Asterisk and # (in panel B) indicate significantly different from sham group (P ≤ 0.05). (**A**) D_A_ was increased in 3 days, 7 days, and 14 days groups compared to sham group. (**B**) D_V_ was increased in 3 days and 7 days groups compared to sham group. V_V_ (interrupted line) was significantly lower in 3 hours, 3 days, 7 days, and 14 days groups compared to sham group. (**C**) TRBF was decreased at 3 hours, 3 days, 7 days and 14 days groups compared to sham group.

The mean and standard deviation of arterial diameter (D_A_), venous diameter (D_V_), venous velocity (V_V_), and TRBF for each group (sham, 3 hours, 3 days, 7 days, and 14 days BCCAO) are displayed in Fig. 2. D_A_, D_V_, V_V,_ and TRBF of the sham group were 38 ± 3 µm, 48 ± 4 µm, 12.7 ± 2.0 mm/sec, and 7.7 ± 2.4 µL/min, respectively.

Compared to the sham group, D_A_ and D_V_ were not significantly different after 3 hours of BCAAO (P ≥ 0.36). Differences between groups as estimated by the statistical model are presented by the symbol β. D_A_ and D_V_ were higher after 3 days (β = +10 µm and +12 µm, respectively) and 7 days (β = +19 µm and +13 µm, respectively) (P ≤ 0.02). D_A_ was also higher (β = +15 µm) (P < 0.001), while D_V_ was not significantly different (P = 0.46) after 14 days of BCCAO.

Compared to the sham group, V_V_ was significantly lower after 3 hours (β = −10.5 mm/sec), 3 days (β = −10.1 mm/sec), 7 days (β = −7.4 mm/sec), and 14 days of BCCAO (β = −7.0 mm/sec) (P < 0.001).

TRBF was decreased after 3 hours (β = −6.4 µL/min), 3 days (β = −5.3 µL/min), 7 days (β = −2.6 µL/min), and 14 days of BCCAO (β = −3.4 µL/min) compared to the sham group (P ≤ 0.02). There was no significant difference in TRBF between 3 hours and 3 days of BCCAO (P = 0.07) or between 7 days and 14 days of BCCAO (P = 0.49). However, TRBF was increased between 3 days and 7 days of BCCAO (P = 0.01).

### Vascular oxygen content

**Figure 3:**
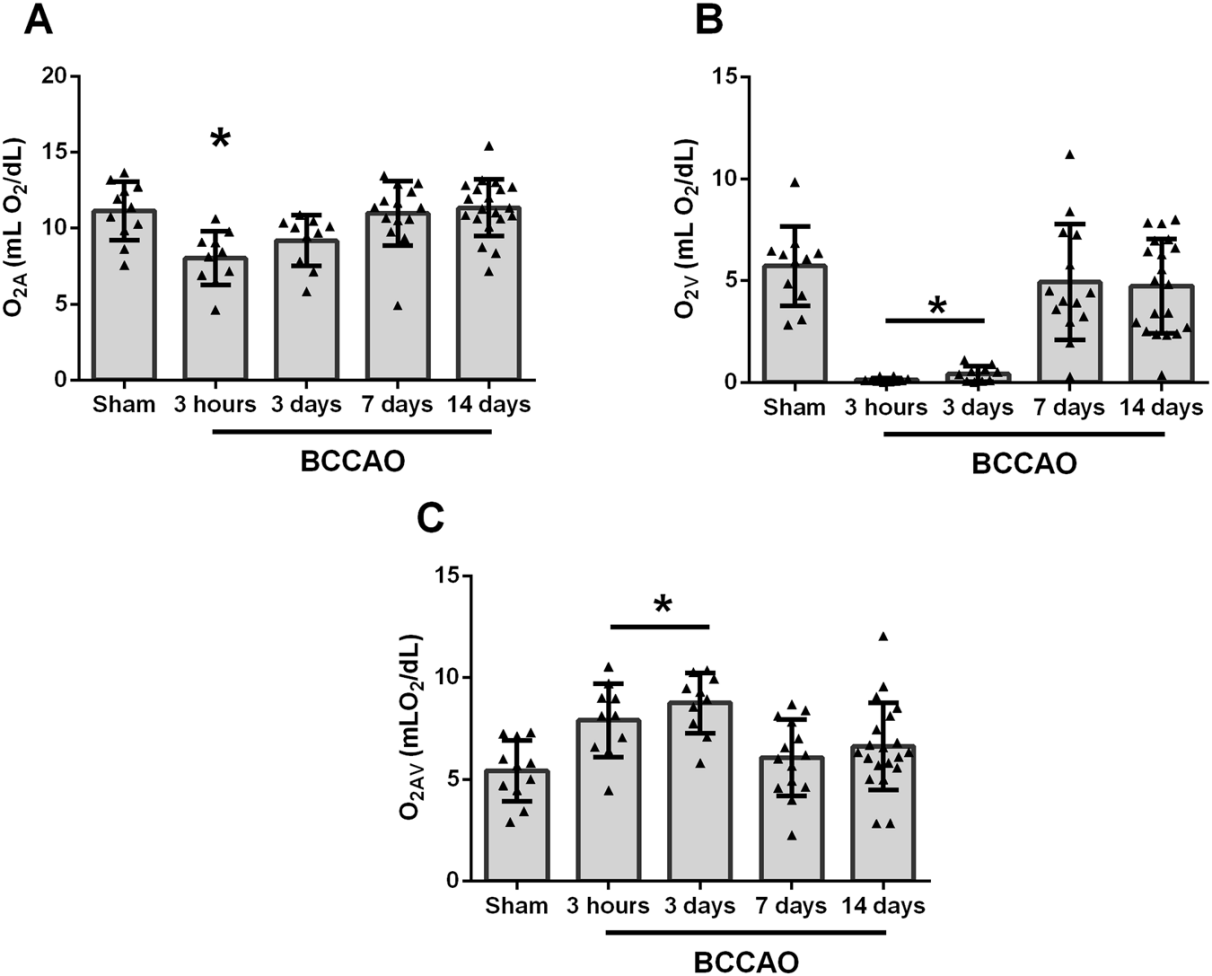
Comparison of arterial oxygen content (O_2A_), venous oxygen content (O_2V_), and arterio-venous oxygen content difference (O_2AV_) measured 3 hours, 3 days, 7 days, and 14 days after bilateral common carotid artery occlusion (BCCAO) as well as in sham group. Error bars indicate standard deviations. Asterisk indicates significantly different than sham group (P ≤ 0.05). (**A**) O_2A_ was significantly lower in 3 hours compared to sham group. (**B**) O_2V_ was significantly lower in 3 hours and 3 days groups compared to sham group. (**C**) O_2AV_ was significantly higher in 3 hours and 3 days groups compared to sham group.

The mean and standard deviation of arterial oxygen content (O_2A_), venous oxygen content (O_2V_), and arteriovenous oxygen difference (O_2AV_) for each group are presented in Fig. 3. O_2A_, O_2V_, and O_2AV_ of the sham group were 11.2 ± 1.9 mLO_2_/dL, 5.7 ± 1.9 mLO_2_/dL, and 5.4 ± 1.5 mLO_2_/dL, respectively. O_2A_ was lower after 3 hours of BCCAO (β = −3.1 mLO_2_/dL) compared to the sham group (P = 0.005). Likewise, O_2V_ was lower after 3 hours (β = −5.7 mLO_2_/dL) and 3 days of BCCAO (β = −5.4 mLO_2_/dL) (P < 0.001). Accordingly, O_2AV_ was higher after 3 hours (β = +2.5 mLO_2_/dL) and 3 days of BCCAO (β = + 3.4 mLO_2_/dL) compared to the sham group (P ≤ 0.01).

### Oxygen metabolism, oxygen delivery, and oxygen extraction fraction

**Figure 4:**
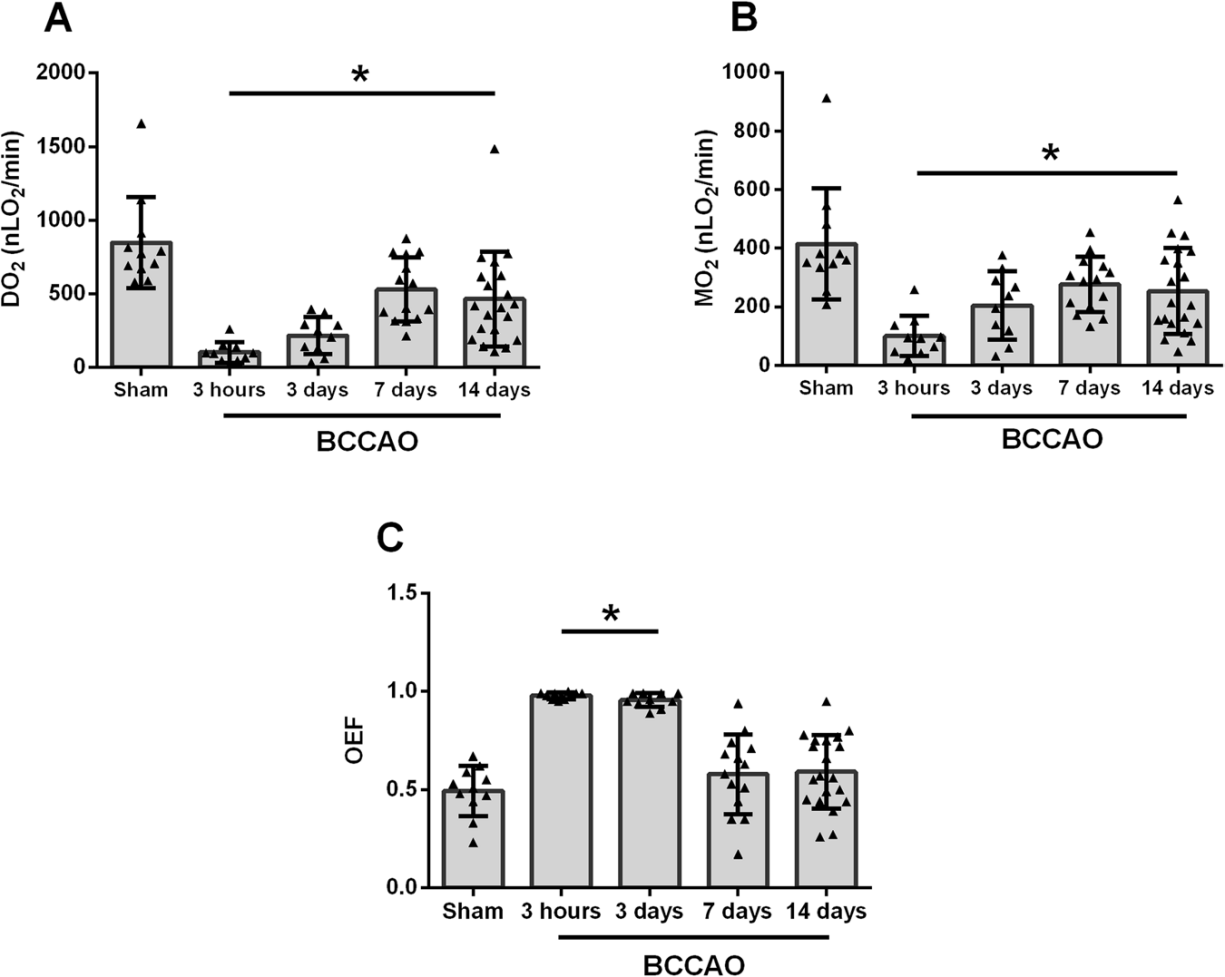
Comparison of oxygen delivery (DO_2_), oxygen metabolism (MO_2_), and oxygen extraction fraction (OEF) measured 3 hours, 3 days, 7 days, and 14 days after bilateral common carotid artery occlusion (BCCAO) as well as in sham group. Error bars indicate standard deviations. Asterisk indicates significantly different than sham group (P ≤ 0.05). (**A**) DO_2_ was significantly lower in 3 hours, 3 days, 7 days, and 14 days groups compared to sham group (**B**) MO_2_ was significantly lower in 3 hours, 3 days, 7 days, and 14 days groups compared to sham group. (**C**) OEF was significantly higher in 3 hours and 3 days groups compared to sham group.

The mean and standard deviation of oxygen metrics (DO_2,_ MO_2_, and OEF) for each group are presented in Fig. 4. OEF was calculated as the ratio of MO_2_ to DO_2_ or alternatively as the ratio of O_2AV_ to O_2A_^18^.

DO_2_ was 848 ± 311 nLO_2_/min in the sham group. Compared to the sham group, DO_2_ was decreased after 3 hours (β = −745 nLO_2_/min), 3 days (β = −624 nLO_2_/min), 7 days (β = −312 nLO_2_/min), and 14 days of BCCAO (β = −383 nLO_2_/min) (P ≤ 0.01). There was no significant difference in DO_2_ between 3 hours and 3 days of BCCAO (P = 0.06) or between 7 days and 14 days of BCCAO (P = 0.57). However, DO_2_ was increased between 3 days and 7 days of BCCAO (P = 0.005).

MO_2_ was 415 ± 190 nLO_2_/min in the sham group. Compared to the sham group, MO_2_ was decreased after 3 hours (β = −307 nLO_2_/min), 3 days (β = −196 nLO_2_/min), 7 days (β = −135 nLO_2_/min), and 14 days of BCCAO (β = −155 nLO_2_/min) (P ≤ 0.04). There was no significant difference in MO_2_ between 3 hours and 3 days of BCCAO (P = 0.07), between 3 days and 7 days of BCCAO (P = 0.36), or between 7 days and 14 days of BCCAO (P = 0.77).

OEF was 0.49 ± 0.13 in the sham group. Compared to the sham group, OEF was increased after 3 hours (β = +0.50) and 3 days (β = +0.47) (P < 0.001) but was not significantly different than the sham group after 7 days or 14 days of BCCAO (P ≥ 0.14). There was no significant difference in OEF between 3 hours and 3 days of BCCAO (P = 0.09) or between 7 days and 14 days of BCCAO (P = 0.83). However, OEF was decreased between 3 days and 7 days of BCCAO (P = 0.002).

## Discussion

For the first time alterations in the ability of the retinal vasculature to deliver oxygen and the retinal tissue to utilize oxygen were shown by the evaluation of oxygen metrics at several time points over long durations of BCCAO. We demonstrated reduced DO_2_ and MO_2_ up to 14 days after BCCAO. However, there was no progressive decrease in MO_2_, leading us to reject our hypothesis. This is likely because the partial reduction of blood flow allowed continued survival of some cells following the initial insult and irreversible injury to other cells. Furthermore, by 7 to 14 days MO_2_ had stabilized at a reduced level and DO_2_ had reached a corresponding reduced value such that OEF approximated the normal value.

Both D_A_ and D_V_ increased after 3 days and 7 days of BCCAO, indicating vasodilation of major retinal vessels in response to BCCAO. Consistent with findings of the current study, a previous study found increased retinal arterial diameter following elevation of intraocular pressure (IOP) in humans^19^ due to adaptation of the vessels to the momentary metabolic requirements of cells causing vasodilation to compensate for reduction in perfusion pressure as a form a vascular autoregulation. However, in contrast to the finding of the current study, increased IOP also resulted in decreased venous diameter^19^. After 14 days of BCCAO, D_A_ remained elevated, while D_V_ was not different than the sham group. Normalization of D_V_ suggests that the retinal vasculature and tissue may have reached a new, reduced metabolic steady state, likely because of an increase in the number of metabolically inactive or lost cells after long-term hypoperfusion due to BCCAO.

In the current study, both TRBF and DO_2_ were reduced up to 14 days after BCCAO, similar to findings of our previous study performed immediately following BCCAO^16^. The BCCAO model causes an abrupt and permanent decrease of blood flow in both the retinal and choroidal circulations and thus resembles human ophthalmic artery occlusion and ocular ischemic syndrome. However, it differs from other human conditions in which only the retinal circulation is involved, such as retinal artery occlusions and diabetic retinopathy. The presence of blood flow during BCCAO may be possible by retrograde flow through the distal internal carotid artery from the Circle of Willis and then orthograde via the pterygopalatine artery (Blair *et al*., unpublished data). The observed compensatory dilation of major retinal vessels accounts for the measured increase in both TRBF and DO_2_ from 3 days to 7 days. The vascular compensatory response is presumably due to enlargement of vertebral and basilar arteries, which feed the circle of Willis^20^.

MO_2_ was reduced at all time points between 3 hours and 14 days after BCCAO, consistent with previously reported findings of functional impairments shown by ERG^4–6^ and PLR^7–10^. Threshold values for rates of MO_2_ have been reported that correlate well with brain tissue survival, and they appear to be superior to OEF and other parameters for predicting outcome^21–24^. Future longitudinal studies are needed to establish MO_2_ thresholds for retinal tissue survival under ischemic conditions.

Under conditions of reduced blood flow up to 3 days after BCCAO, OEF essentially approximated its maximum value of 1, along with extremely low values of O_2V_, which indicates inadequate oxygen availability to meet the tissue’s demand. In the brain, experimental studies have shown that elevation of OEF is associated with threatened tissue^25^. It has been proposed that with misery perfusion, in which blood flow is reduced relative to the regional metabolic demand for oxygen^26^, along with maximized OEF, cellular dysfunction or injury can occur at 2 levels of severity: first, ischemic hypoxia, in which cells adapt to low tissue oxygenation and maintain structural integrity, and second, ischemic anoxia, in which metabolism stops and complex metabolic cascades leading to cell death have been initiated^27^. In hypoxic neural tissue, electrical activity and function may be restored through prompt restoration of blood flow^27–29^. This salvageable, hypoxic tissue at risk for irreversible cell death is classified as penumbra^27^. The retina is considered a part of the central nervous system (CNS), and although morphologies of RGCs and CNS neurons differ to some extent, they have similar properties^30^. Therefore, it is possible that during BCCAO some retinal cells may have existed in a state of penumbra, such that their function may be potentially recovered with timely restoration of blood flow.

Normalization of OEF coupled with reduced DO_2_ and MO_2_ after 7 days and 14 days of BCCAO suggests that the oxygen metabolic demand of the tissue had diminished due to anoxic conditions resulting in cell death. Since cells located farthest from the capillaries will have the least oxygen supply^28,29^, they likely will die first, while cells closer to capillaries may be able to survive. The reduced MO_2_ measured after one week of BCCAO represents the net metabolism of the remaining living cells, and DO_2_ had reached a corresponding reduced value such that OEF normalized.

The current study had some limitations. First, the systemic physiology of the animals was not monitored during imaging. Although the same anesthesia protocol was used for all animals, there may have been some inter-animal variations conditions. Second, the measured responses to ischemia may be dependent on the duration of anesthesia and age. The observed increase in DO_2_ between 3 days and 7 days after BCCAO was not detected in related studies conducted under a longer duration of anesthesia in rats of different ages^17^. Third, the effect of BCCAO on choroidal circulation and recovery of retinal function which depends on changes in both retinal and choroidal hemodynamics were not evaluated in the current study. Fourth, reduced MO_2_ may have been in part caused by reduced oxygen extraction from the retinal blood supply, which can occur by an increase in oxygen delivery from the choroidal circulation due to lower consumption or death of photoreceptors induced by the ischemic insult. Accordingly, the findings may not be generalizable to other groups with different strains, species, age, sex, or anesthesia durations. Fifth, anesthesia may have caused systemic hypoxia in the sham group, resulting in vasodilation and increased blood flow compared to non-anesthetized condition, and hence TRBF recovery in the study groups may have been underestimated. However, since all groups of rats were under similar physiological conditions, this factor minimally affected the direction of reported changes. Finally, the findings were based on a cross sectional study and future longitudinal studies are needed to characterize the time course of changes in oxygen metrics in the same animal.

In conclusion, sustained impairments of DO_2_ and MO_2_ were demonstrated up to 14 days after BCCAO. Additionally, OEF was increased initially after BCCAO, but with longer durations of ischemia, DO_2_ stabilized at a value such that the ratio between MO_2_ and DO_2_, that is, OEF, approximated the normal value. These findings contribute to better understanding of the pathophysiology of retinal ischemic injury that may be necessary for development and testing of therapeutic interventions for retinal ischemia.

## Materials and Methods

### Animals

All procedures were approved by the University of Southern California Institutional Animal Care and Use Committee and adhered to the articles of the statement of Use of Animals in Ophthalmic and Vision research by the Association for Research in Vision and Ophthalmology. The experiments have been reported following the Animal Research: Reporting *in Vivo* Experiments guidelines. The study was performed in 35 adult (age: 12–20 weeks) male Long-Evans rats (weight: 240–520 g) (Charles River, San Diego, CA). Twenty-nine rats were subjected to permanent BCCAO and imaged after 3 hours (N = 5), 3 days (N = 6), 7 days (N = 8), or 14 days (N = 10). Six rats underwent a sham procedure and were imaged after 3 days (N = 3) or 14 days (N = 3). Five rats died before images could be acquired and were not included in the experimental data.

Rats were acclimated for 3 days before being subjected to random grouping of cohorts. They were kept under environmentally controlled conditions with a 12-hour/12-hour light/dark cycle at 20–22 °C, were fed a standard rat chow diet, and had free access to food and water. For BCCAO procedure, anesthesia was administered with 1.5–2.5% isoflurane, balance oxygen. The common carotid arteries were accessed via a midline prelaryngeal incision, and cleanly dissected from the sympathetic and vagus nerves. Silk sutures (5–0 gauge) were used to completely ligate both common carotid arteries, leaving blood flow to the eye from other pathways, likely retrograde through the distal internal carotid artery from the Circle of Willis and then orthograde via the pterygopalatine artery (Blair *et al*., unpublished data). Sham groups underwent the same procedure, but without ligation of the carotid arteries. For imaging, rats were anesthetized with intraperitoneal injections of Ketamine (90 mg/kg) and Xylazine (5 mg/kg). Additional doses were given as needed. Prior to imaging, a catheter was placed in the femoral artery for delivery of 2-µm polystyrene fluorescent microspheres (Life Technologies, Eugene, OR) at a concentration of 10^7^ particles/mL and Pd-Porphine (Frontier Science, Boston, MA) at a dosage of 20 mg/kg. Pupils were dilated with 2.5% Phenylephrine (Paragon, Portland, OR) and 1% Tropicamide (Bausch and Lomb, Tampa, FL). Rats were placed on a water circulating heated holder for imaging. A glass cover slip with 2.5% hypromellose ophthalmic demulcent solution (HUB Pharmaceuticals, Plymouth, MI) was applied to the cornea to maintain hydration and eliminate its refractive power. Imaging was performed in both eyes. Personnel who conducted the experiments were knowledgeable of the group allocation during the imaging sessions.

### Blood flow imaging

Venous blood velocity (V) and diameter (D) were measured by our previously described imaging system^31,32^. For D measurements, the light illumination of a slit lamp biomicroscope coupled with a green filter (540 ± 5 nm) was used to capture red-free retinal images. Registered mean images were analyzed to determine the vessel boundaries based on the full width at half maximum of intensity profiles perpendicular to the vessel centerline at several consecutive locations along each vessel^31,32^. Measurements in individual vessels were averaged to obtain mean D_A_ and D_V_ per eye. For V measurements, a 488-nm diode excitation laser and an emission filter (560 ± 60 nm) were used to acquire 520 fluorescence images at 108 Hz. Image sequences were analyzed to determine the displacement of microspheres along each vein segment over time^31,32^. Measurements in individual veins were averaged to calculate a mean V_V_ per eye. Blood flow was calculated in each vein as VπD^2^/4 and then summed over all the veins to calculate a total retinal blood flow (TRBF) per eye.

### Vascular PO_2_ Imaging

Retinal vascular PO_2_ was measured using our established optical section phosphorescence lifetime imaging system^33^. A vertical laser line (532 nm) was projected on the retina at an angle and an infrared filter with a cutoff wavelength of 650 nm was placed in the imaging path. Phosphorescence lifetimes of Pd-Porphine within all major retinal arteries and veins were determined using a frequency-domain approach and converted to PO_2_ measurements using the Stern-Volmer equation^34,35^. Three PO_2_ measurements were averaged for each vein and artery.

### Oxygen delivery, metabolism, extraction fraction

The oxygen (O_2_) content of the retinal blood vessels was determined as the sum of oxygen bound to hemoglobin and dissolved in blood^36^: O_2_ content = SO_2_ × HgB × C + k × PO_2_, where SO_2_ is the oxygen saturation calculated from the rat hemoglobin dissociation curve using measured PO_2_ and blood pH values from literature, HgB is the rat hemoglobin concentration value (13.8 g/dL)^37^, C is the maximum oxygen-carrying capacity of hemoglobin (1.39 mL O_2_/g)^38^, and k is the solubility of oxygen in blood (0.0032 mL O_2_/dL mmHg)^39^. Mean O_2A_ and O_2V_ were calculated by averaging values in all arteries and veins, respectively. O_2AV_ was computed as the difference between O_2A_ and O_2V_. In each eye, DO_2_, MO_2_, and OEF were calculated as: TRBF × O_2A_, TRBF × O_2AV,_ and MO_2_/DO_2_, respectively. Alternatively, OEF can by calculated as the ratio of O_2AV_ to O_2A_, without measurements of TRBF, as demonstrated previously^18^.

## Data analysis

Statistical analyses were performed using SSPS Statistics, Version 24 (IBM Armonk, New York). Since there was no statistically significant difference in oxygen metrics (DO_2_, MO_2_, OEF, and TRBF) among the sham groups (3 days and 14 days) by mixed linear model analysis, data in both sham groups were combined to generate a single sham group. Data in both eyes were classified into 5 groups according to duration of BCCAO (3 hours, 3 days, 7 days, 14 days) or sham. Compiled O_2A_, O_2V_ and TRBF data were evaluated by group and 5 outliers (values beyond 3 times the interquartile range) were removed, leaving data in a total of 65 eyes: 3 hours (N = 10 eyes), 3 days (N = 10 eyes), 7 days (N = 14 eyes), 14 days (N = 20 eyes) BCCAO groups and sham group (N = 11 eyes). Given an effect size of 0.5, to detect DO_2_ differences among groups with 80% power and alpha = 0.05, a sample size of 11 is needed. Oxygen metrics were compared among groups by mixed linear models with group and eye as fixed effects and animal as a random effect. The models generated estimated differences (β) between groups. Statistical significance was accepted at P ≤ 0.05.

## Data Availability

Data are available upon request to the corresponding author.
